# Radiographic signs of osteoarthritis in the temporomandibular joint among young adults and associated imaging factors: A cone-beam computed tomography study

**DOI:** 10.4317/jced.63580

**Published:** 2025-12-30

**Authors:** Soledad Jimena Juarez-Pacheco, Karla Franciela Lopez-Ramírez, Jhoana Mercedes Llaguno-Rubio, Gustavo Adolf Fiori-Chincaro, Luis Ernesto Arriola-Guillén

**Affiliations:** 1 Division of Oral and Maxillofacial Radiology, Faculty of Dentistry, Centro Universitário do Norte de São Paulo (UNORTE), São Paulo, Brazil. ORCID ID: 0009-0007-7562-5020; 2 Division of Oral and Maxillofacial Radiology, Faculty of Dentistry, Centro Universitário do Norte de São Paulo (UNORTE), São Paulo, Brazil. ORCID ID: 0009-0002-3372-6636; 3 Associate Professor, Division of Oral Radiology, Instituto Latinoamericano de Altos Estudios en Estomatología (ILAE), Lima, Peru. ORCID ID: 0000-0002-0223-608X; 4 Associate Professor, Division of Oral Radiology, Instituto Latinoamericano de Altos Estudios en Estomatología (ILAE), Lima, Peru. ORCID ID: 0000-0001-9273-8587; 5 Ph.D. and Associate Professor, Division of Orthodontics, Faculty of Dentistry, Universidad Científica del Sur, Lima, Peru. ORCID ID: 0000-0003-0010-5948

## Abstract

**Background:**

This study aims to determine the prevalence of radiographic signs of osteoarthritis in the Temporomandibular Joint (TMJ) among young adults and to identify the associated imaging factors using Cone-Beam Computed Tomography (CBCT) in a population from Arequipa, Peru.

**Material and Methods:**

This cross-sectional study involved the analysis of 311 CBCT scans from young adults aged 18 to 40 years. To ensure high image quality and comprehensive clinical data, we applied specific inclusion and exclusion criteria in the study. We evaluated radiographic signs of condylar osteoarthritis, which included flattening, erosion, sclerosis, osteophytes, and subchondral cysts. Additionally, we examined several predictor factors, including sex, age, and two associated imaging factors such as edentulism, and occlusal wear. Furthermore, examiner training and calibration were conducted with the Kappa concordance test. We performed descriptive, bivariate, and multivariate statistical analyses using the chi-square test and binary logistic regression (p&lt;0.05).

**Results:**

The prevalence of condylar changes indicative of osteoarthritis was significantly high. Flattening was observed in 30.2% of cases, erosion in 16.1%, and sclerosis in 10.3%. There was a notable association between condylar pathologies and sex; specifically, a significant association was observed between condylar sclerosis and female sex, occurring in 14.1% of women compared to 4.2% of men (p = 0.003). Furthermore, both age (Exp(B) = 1.071; p = 0.001) and edentulism (Exp(B) = 4.353; p &lt; 0.001) significantly influenced the presence of condylar flattening. Similarly, age (Exp(B) = 1.050; p = 0.041) and edentulism (Exp(B) = 2.630; p = 0.024) also affected condylar erosion. Additionally, the occurrence of condylar sclerosis was influenced by age (Exp(B) = 1.067; p = 0.044) and edentulism (Exp(B) = 4.276; p = 0.008).

**Conclusions:**

The findings suggest that temporomandibular osteoarthritis can develop moderately in early adulthood, primarily characterized by condylar flattening, erosion, and sclerosis. The data indicate that certain predictor variables, such as sex, reveal that condylar sclerosis is more commonly observed in females. Additionally, age and edentulism are significant factors linked to changes in the condyle, including condylar flattening, erosion, and sclerosis.

## Introduction

Temporomandibular joint (TMJ) osteoarthritis is a degenerative condition characterized by the degradation of cartilage, changes in subchondral bone, and morphological alterations of the condyle, which may lead to pain, joint noises, functional limitations, and a reduced quality of life (1 , 2). Traditionally, degenerative bone changes have been associated with middle-aged and older individuals; however, recent studies have indicated that young adults may also exhibit radiographic signs of condylar osteoarthritis, even in the absence of significant clinical symptoms. A retrospective study involving adults aged 18 to 30 years found that approximately 39.9% showed bony alterations in both condyles, while up to 64.3% displayed changes on at least one side. The most common findings were condylar erosion, flattening, and osteophyte formation (3). Furthermore, factors such as posterior tooth loss, occlusal imbalance, parafunctional habits, and systemic variables such as hormonal influences-particularly in women-have been observed to play a role in the etiopathogenesis of TMJ osteoarthritis (4 - 8). However, most studies have focused on older populations or on patients with evident symptoms of temporomandibular disorders (TMD), leaving unclear how early these lesions appear in asymptomatic young individuals and how local factors are associated with detectable alterations on advanced imaging techniques. The use of cone-beam computed tomography (CBCT) has significantly advanced the field, as this three-dimensional imaging technique provides a more precise visualization and classification of changes in condylar bone. These changes, which include flattening, erosion, sclerosis, osteophytes, and subchondral cysts, are often not detectable on panoramic or transcranial radiographs (9 - 11). Moreover, few studies have focused on young populations under strict inclusion and exclusion criteria, and even fewer have quantified the specific impacts of tooth loss and parafunctional habits. Additionally, there is a limited understanding of the relationship between radiographic findings and clinical function in these groups. Therefore, this study aims to determine the prevalence of TMJ osteoarthritis among young adult patients in a Peruvian population. It also seeks to identify potential associated factors by evaluating CBCT images to detect morphological alterations in the temporomandibular joint. This approach has the potential to assist in early diagnosis and improve our understanding of disease progression within this population.

## Material and Methods

**Figure 1:**
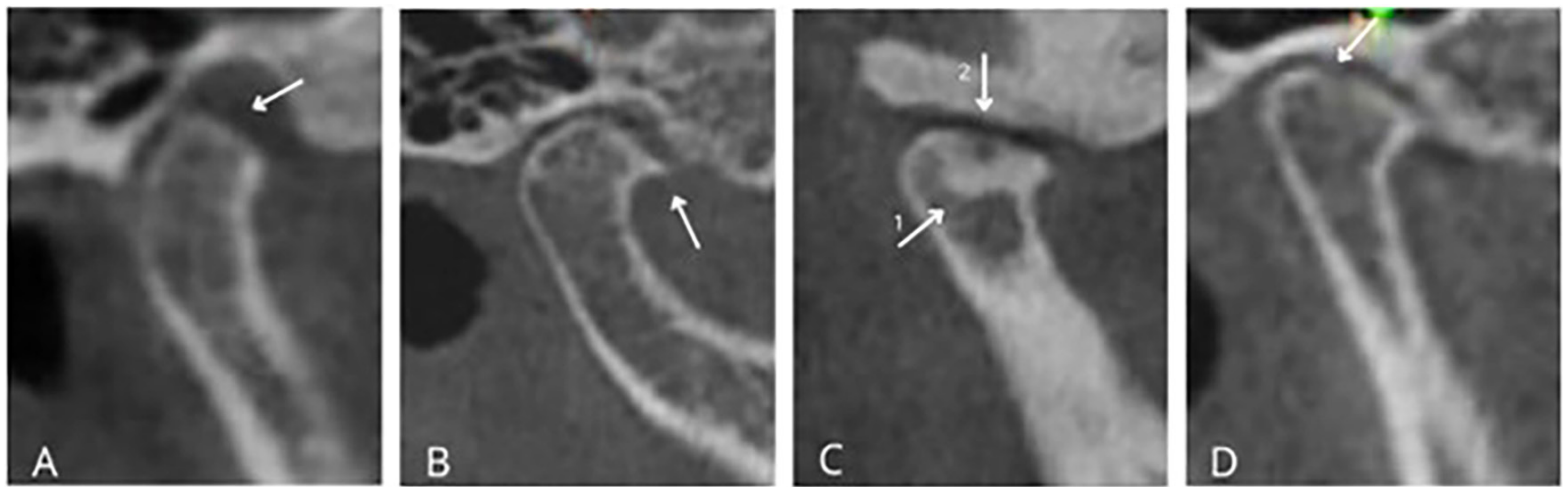
Radiographic signs of condylar osteoarthritis. A. Flattening; B. Osteophyte; C. (1) Sclerosis, (2) Cyst; D. Erosion.

This cross-sectional and retrospective study was approved by the Ethics Committee of Universidad Católica de Santa María in Arequipa, Peru, under protocol number (257 - 2025 CIEI-UCSM). The sample comprised 311 cone-beam computed tomography (CBCT) scans, which were randomly selected from a database of 2,354 records collected between April 2022 and March 2025 at the Radiological Center of the Faculty of Dentistry, Universidad Católica de Santa María, Arequipa, Peru. The study specifically included CBCT scans from patients aged 18 to 40 years who had complete data on sex and age. Scans that contained artifacts were of poor quality, or showed bone pathologies that hindered evaluation were excluded from the study. The variables analyzed included radiographic signs of condylar osteoarthritis, such as flattening, erosion, sclerosis, osteophytes, subchondral cysts, and loose bodies (Table 1, Fig. 1).

Table 1

1

**Figure 2:**
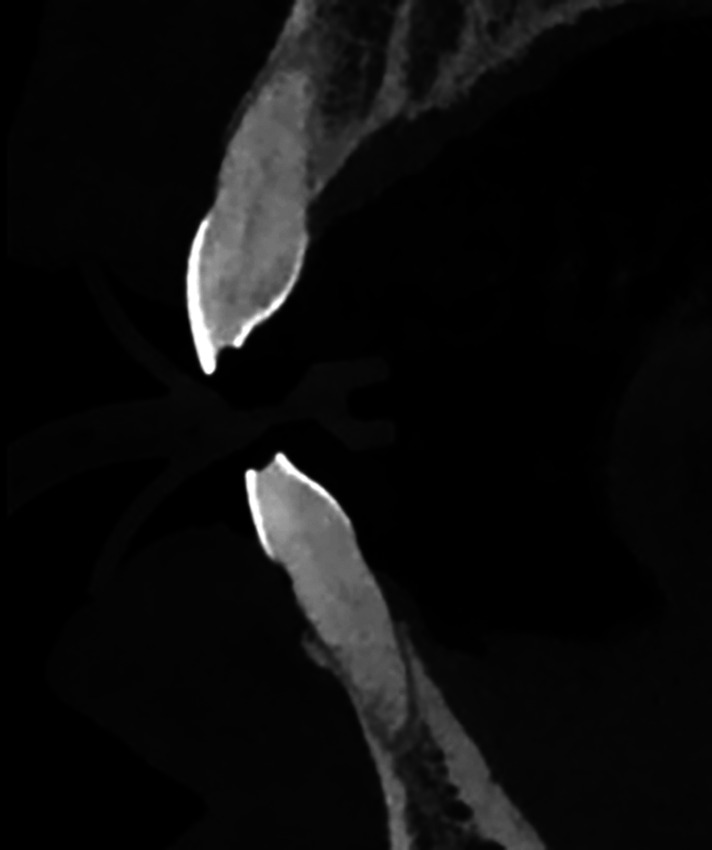
Occlusal wear visualization on CBCT.

Additionally, factors related to dental imaging, such as occlusal wear and tooth loss (edentulism), were also taken into consideration. (Table 1, Fig. 2) All these measurements were made using three-dimensional slices on the tomographies.

2

All measurements were conducted by two examiners who were trained and calibrated through a pilot test. The intra-examiner and inter-examiner agreement achieved a Kappa coefficient greater than 0.9 for both examiners. - Statistical analysis Data analysis was performed using IBM SPSS Statistics version 25.0 (IBM Corp., Armonk, NY, USA). Descriptive and comparative statistics were utilized, employing Chi-square tests and Fisher's exact tests. To identify predictive factors associated with condylar changes, we conducted a binary logistic regression analysis. A significant level of 5% (p &lt; 0.05) was established, and the results are presented as Exp(B) along with 95% confidence intervals.

## Results

Table 2 presents the demographic characteristics of the sample, which includes 119 men and 192 women, with an average age of 27.96 years for men and 30.10 years for women.

Table 2

Table 3 highlights the associations between condylar pathologies and sex, revealing a significant correlation only between the presence of condylar sclerosis and the female sex, occurring in 14.1% of women compared to 4.2% of men.

Table 3

This finding was determined using Fisher's exact test (p = 0.003). The most prevalent pathologies identified were condylar flattening (30.2%) and erosion (16.1%), while condylar cysts were the least frequent, occurring in only 0.6% of cases, with no observations of loose bodies (0%). Table 4 summarizes the results of binary logistic regression analyses conducted to assess the influence of predictive variables on condylar pathologies.

Table 4

Age (Exp(B) = 1.071; p = 0.001) and edentulism (Exp(B) = 4.353; p &lt; 0.001) were found to influence the presence of condylar flattening significantly. In addition, condylar erosion was influenced by age (Exp(B) = 1.050; p = 0.041) and edentulism (Exp(B) = 2.630; p = 0.024). Lastly, the occurrence of condylar sclerosis was also significantly influenced by age (Exp(B) = 1.067; p = 0.044) and edentulism (Exp(B) = 4.276; p = 0.008).

## Discussion

The results of this study reveal a significant prevalence of condylar alterations-primarily flattening, erosion, and sclerosis-in young adults. This finding confirms that temporomandibular joint (TMJ) osteoarthritis is not exclusive to older age groups. Our results align with Latin American studies indicating early degenerative changes in individuals under 40 years old, suggesting that functional load and parafunctional habits may be more influential than chronological age in the development of these lesions (3 , 9 , 10). Specifically, our study found a significant association between condylar sclerosis and female sex, with 14.1% of women affected compared to only 4.2% of men (p = 0.003). However, we did not observe any associations with other condylar pathologies, which contrasts with findings from different studies. This discrepancy may be attributed to the specific nature of our sample, as only severe conditions, such as sclerosis, are more likely to show such associations. The higher prevalence of condylar sclerosis in women highlights the influence of endocrine factors on joint pathophysiology. Numerous studies have documented the modulatory role of estrogens in bone metabolism and cartilage homeostasis (8 , 16). Variations in estrogen receptors may increase susceptibility to degenerative changes, which could explain the trend we observed in our study. Clinically, this finding emphasizes the need to consider hormonal variations as a differential risk factor when evaluating and monitoring young women with temporomandibular dysfunction. Additionally, our findings indicated that age and tooth loss were significant predictors of condylar flattening, erosion, and sclerosis. These results support previous research linking edentulism (loss of teeth) to increased biomechanical stress on the temporomandibular joint (TMJ) due to abnormal redistribution of masticatory loads (12). This functional alteration can lead to repetitive microtraumas and joint overload, which initially trigger adaptive bone remodeling processes but may ultimately progress to degenerative changes. Clinical and radiographic studies have shown that the absence of posterior dental support modifies condylar position and compromises joint function, promoting structural deterioration (5 , 13). Moreover, recent research indicates that tooth loss is associated with condylar displacement and variations in joint space-conditions that may precede and facilitate the onset of erosions and sclerosis, reinforcing the idea that edentulism is a potentially modifiable risk factor (14 , 15). Regarding diagnosis, cone-beam computed tomography (CBCT) has proven highly effective for detecting both early and advanced structural changes in the condyle. Consistent with previous studies (3 , 16 , 17), CBCT enabled the identification of subtle bone microchanges that are not visible on panoramic or transcranial radiographs, confirming its role as the preferred imaging modality for morphological evaluation of the TMJ. However, the literature emphasizes that the correlation between imaging findings and clinical symptoms may be weak, underscoring the importance of integrating radiographic assessments with comprehensive clinical examinations to achieve more accurate interpretations. The clinical implications of these findings indicate that early identification of degenerative signs in young adults may impact on therapeutic strategies in orthodontics, rehabilitation, and restorative dentistry. By incorporating advanced imaging diagnostic protocols into clinical practice, we can help prevent the progression of degenerative lesions. This can be achieved through opportunely occlusal support rehabilitation, correction of parafunctional habits, and multidisciplinary management of joint load. Furthermore, these findings emphasize the importance of preventive education regarding balanced masticatory function and tooth preservation, which serve as protective factors for the condyle-disc complex. Although this study used a three-dimensional method to improve diagnosis and included a large sample of CT scans, the findings cannot be generalized, and further research in other racial groups is necessary. Finally, this study's findings indicate that radiographic signs of temporomandibular osteoarthritis are common among young adults, confirming that the condition has an early onset and could be influenced by multiple factors. The most significant determinants of changes in the condyle were age and edentulism, while an association was observed between condylar sclerosis and females. Cone-beam computed tomography (CBCT) has proven to be an effective diagnostic tool for identifying early changes in bone structure. It can help guide personalized preventive and therapeutic interventions aimed at preserving joint function and improving the quality of life for young patients.

## Conclusions

The prevalence of temporomandibular osteoarthritis is moderate during early adulthood. This condition is characterized primarily by condylar flattening, erosion, and sclerosis. Data indicate that certain predictor variables, such as sex, show that condylar sclerosis is more common in females. Additionally, age and edentulism are significant factors associated with changes in the condyle, including condylar flattening, erosion, and sclerosis.

## Figures and Tables

**Table 1:** Radiographic Signs of Osteoarthritis and associated factors.

Radiographic Sign	Definition	
Flattening	Loss of the surface rounded contour.	
Erosion	Continuity loss of the articular cortex.	
Sclerosis	Lack of clear trabecular orientation with no delineation between the cortical layer and the trabecular bone that extends throughout the condylar head.	

Osteophytes	Marginal hypertrophy with sclerotic borders and exophytic angular formation of osseous tissue arising from the surface.	

Cyst	Cavity below the articular surface that deviates from normal marrow pattern.	

Loose body	Well-defined calcified structure presenting lack of continuity with the bone components of the joint.	
Occlusal wear	Reduction in the height of dental crowns (incisal edges and cusps)	
Edentulism	Loss of dental pieces	

1

**Table 2:** Characteristics of the study sample.

Sex	n	Mean	SD
Female	192	30.10	8.05
Male	119	27.96	7.57

p=0.020, Student’s t-test

**Table 3:** Association between condylar pathologies and sex.

Sex	Indicator	Flattening of the condyle			p-value
		Absent	Present	Total	
Female	n	128	64	192	0.082
	%	66,7	33,3	100	
Male	n	89	30	119	
	%	74,8	25,2	100	
Total	n	217	94	311	
	%	69,8	30,2	100	
Sex	Indicator	Erosion of the condyle			p-value
		Absent	Present	Total	
Female	n	159	33	192	0.304
	%	82,8	17,2	100	
Male	n	102	17	119	
	%	85,7	14,3	100	
Total	n	261	50	311	
	%	83,9	16,1	100	
Sex	Indicator	Sclerosis of the condyle			p-value
		Absent	Present	Total	
Female	n	165	27	192	0.003*
	%	85,9	14,1	100	
Male	n	114	5	119	
	%	95,8	4,2	100	
Total	n	279	32	311	
	%	89,7	10,3	100	
Sex	Indicator	Osteophytes of the condyle			p-value
		Absent	Present	Total	
Female	n	187	5	192	0.088
	%	97,4	2,6	100	
Male	n	119	0	119	
	%	100	0	100	
Total	n	306	5	311	
	%	98,4	1,6	100	
Sex	Indicator	Cyst of the condyle			p-value
		Absent	Present	Total	
Female	n	190	2	192	0.380
	%	99	1	100	
Male	n	119	0	119	
	%	100	0	100	
Total	n	309	2	311	
	%	99,4	0,6	100	
Sex	Indicator	Loose body of the condyle			p-value
		Absent	Present	Total	
Female	n	192	0	192	---
	%	100	0	100	
Male	n	119	0	119	
	%	100	0	100	
Total	n	311	0	311	
	%	100	0	100	

Fisher’s exact test*Significant

**Table 4:** Binary logistic regression analysis evaluating the influence of predictive variables on condylar pathologies.

Outcome variable	Predictive variables	p-value	Exp(B)	95% C.I. for Exp(B)	
				Lower	Upper
Condylar flattening	Female	-	-	-	-
	Male	0.820	0.936	0.531	1.652
	Age	0.001*	1.071	1.030	1.113
	Dental wear (present)	0.302	0.657	0.295	1.460
	Edentulism (present)	<0.001*	4.353	2.093	9.054
	Constant	<0.001*	0.038		
Condylar erosion	Female	-	-	-	-
	Male	0.644	1,179	,587	2,366
	Age	0.041*	1,050	1,002	1,101
	Dental wear (present)	0.121	1,922	,842	4,388
	Edentulism (present)	0.024*	2,630	1,135	6,093
	Constant	<0.001*	,023		
Condylar sclerosis	Female	-	-	-	-
	Male	0.082	0.386	0.133	1.126
	Age	0.044*	1.067	1.002	1.137
	Dental wear (present)	0.025*	2.958	1.148	7.620
	Edentulism (present)	0.008*	4.276	1.451	12.602
	Constant	<0.001*	0.006		
Condylar Osteophyte	Female	-	-	-	-
	Male	0.995	0.000	0.000	
	Age	0.090	1.550	0.933	2.574
	Dental wear (present)	0.993	24981609.213	0.000	
	Edentulism (present)	0.849	1.299	0.087	19.299
	Constant	0.984	0.000		
Condylar cyst	Female	-	-	-	-
	Male	0.995	0.00	0.00	.
	Age	0.307	1316.00	,777	2229.00
	Dental wear (present)	0.993	6034291427.00	0.00	.
	Edentulism (present)	0.994	2057826539.00	0.00	.
	Constant	0.986	0.000		

*Significant

## Data Availability

The data supporting the findings of this study are available from the corresponding author upon reasonable request.
